# *De novo* genome and transcriptome resources of the Adzuki bean borer *Ostrinia scapulalis* (Lepidoptera: Crambidae)

**DOI:** 10.1016/j.dib.2018.01.073

**Published:** 2018-02-01

**Authors:** B. Gschloessl, F. Dorkeld, P. Audiot, A. Bretaudeau, C. Kerdelhué, R. Streiff

**Affiliations:** a CBGP, INRA, CIRAD, IRD, Montpellier SupAgro, Univ Montpellier, Montpellier, France; b INRA, UMR Institut de Génétique, Environnement et Protection des Plantes (IGEPP), BioInformatics Platform for Agroecosystems Arthropods (BIPAA), Campus Beaulieu, Rennes, France; c INRIA, IRISA, GenOuest Core Facility, Campus de Beaulieu, Rennes, France

**Keywords:** *De novo* assembly, Genome, Transcriptome, Gene prediction, Lepidoptera, Crambidae

## Abstract

We present a draft genome assembly with a *de novo* prediction and automated functional annotation of coding genes, and a reference transcriptome of the Adzuki bean borer, *Ostrinia scapulalis*, based on RNA sequencing of various tissues and developmental stages. The genome assembly spans 419 Mb, has a GC content of 37.4% and includes 26,120 predicted coding genes. The reference transcriptome holds 33,080 unigenes and contains a high proportion of a set of genes conserved in eukaryotes and arthropods, used as quality assessment of the reconstructed transcripts. The new genomic and transcriptomic data presented here significantly enrich the public sequence databases for the Crambidae and Lepidoptera, and represent useful resources for future researches related to the evolution and the adaptation of phytophagous moths. The genome and transcriptome assemblies have been deposited and made accessible *via* a NCBI BioProject (id PRJNA390510) and the LepidoDB database (http://bipaa.genouest.org/sp/ostrinia_scapulalis/).

**Specifications Table** Genome and Transcriptome

**Table:** 

Subject area	Biology
More specific subject area	Lepidoptera, Genomics
Type of data	DNA and cDNA sequence reads, genome assembly and transcript assembly
How data was acquired	Shotgun whole genome and cDNA sequencing using Illumina HiSeq 2000
Data format	Analyzed: *i.e.* raw data and assembled sequences
Experimental factors	Genome: total DNA extraction from male larvae of wild samples
	Transcriptome: total RNA extraction from various tissues, developmental stages and of males and females
Experimental features	Genome: DNA sequencing
	Transcriptome: RNA sequencing of various tissues from eggs to adults, controlled conditions
Data source location	Genome: Amiens, Picardie/France (49°54′0.01′″N, 2°18′0″E)
	Transcriptome: Nadarzin, Poland (52°4′2.05″N, 20°47′33.00″E)
Data accessibility	All raw sequence reads are accessible as NCBI BioProject (id PRJNA390510). The OSCA v1.2 draft genome assembly, the reference transcriptome assembly and automatic functional annotations can be found in the LepidoDB database (http://bipaa.genouest.org/sp/ostrinia_scapulalis/).

**Value of the data**

• The draft genome represents the first available genome assembly for *O. scapulalis*.
• The reference transcriptome of *O. scapulalis* will allow comparative expression studies.
• The new genomic and transcriptomic data enrich the public sequence databases for the Crambidae and Lepidoptera.
• The data represent pangenomic resources for future researches related to the evolution and the adaptation of phytophagous moths.

## 1 Data

The Adzuki bean borer, *Ostrinia scapulalis* (hereafter OSCA), is a palaearctic phytophagous moth feeding on various dicotyledons, including hop (*Humulus lupulus*), mugwort (*Artemisia vulgaris*) and hemp (*Cannabis sativa*) [1]. In Europe, it partly co-occurs with its sibling species, the European corn borer, *Ostrinia nubilalis*, which is a major pest of maize (*Zea mays*). Previous studies demonstrated that *O. scapulalis* and *O. nubilalis* are specialized to their respective host plants [2], [3], [4], [5], [6], [7] and that their genetic divergence is rather low so that they can be considered as sibling species [8]. Yet, a few genomic sequences and rearrangements are much more divergent than the rest of the genomic background [1], [9], [10], [11]. These genomic regions are of particular interest to understand the divergence process between *O. scapulalis* and *O. nubilalis*. To further investigate the host adaptation and divergence between these two sibling species at a pangenomic scale, we have elaborated new genomic and transcriptomic resources consisting of an OSCA draft genome and a related reference transcriptome. The latter extends a published transcriptomic set generated with Roche 454 sequencing technology [12].

## 2 Experimental design, materials and methods

### 2.1 *De novo* draft genome

**Fig. 1:**
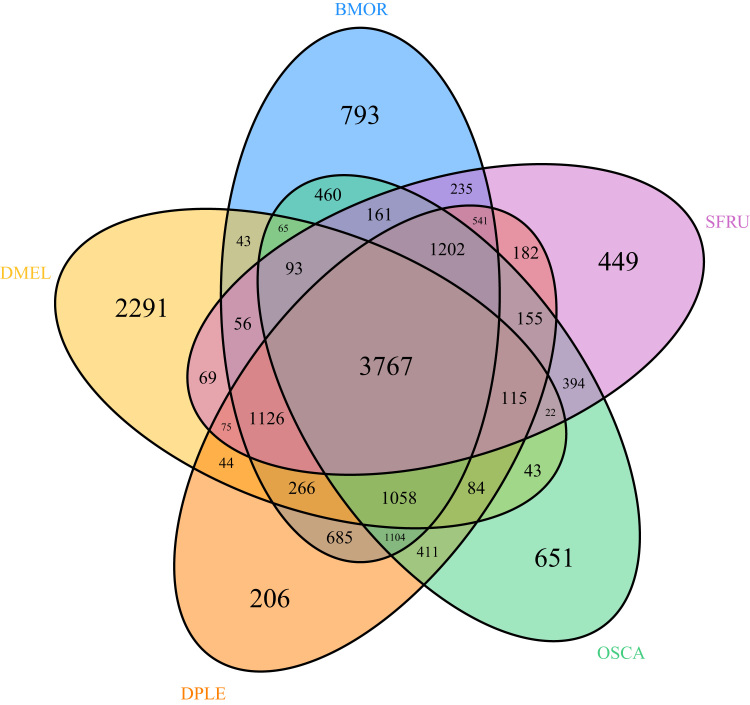
Venn diagram showing all OrthoMCL ortholog groups among the MAKER-predicted *O. scapulalis* proteins (OSCA) and the proteomes of *Spodoptera frugiperda* (SFRU), *Bombyx mori* (BMOR), *Danaus plexippus* (DPLE) and *Drosophila melanogaster* (DMEL).

Diapausing larvae were collected in mugwort stems in 2008 near Amiens (Picardie, France) and stored in 95% ethanol at −20 °C. Whole genomic DNA extracts were obtained from a CTAB-based method [13]. DNA quality and integrity was evaluated through migration on an agarose gel and nanodrop technology. The sex of each sampled larvae was determined with a molecular coamplification of markers specific to each heterochromosome (Z and W in Lepidoptera) as described in Orsucci et al. [6]. Only samples of the ZZ homogametic sex (males in Lepidoptera), were retained for the libraries construction. A 2 × 100 bp shot-gun paired-end library and a 3 (2 × 50 bp) and an 8 kb (2 × 100 bp) mate-pair library were generated using the DNA extract of one larva for each library and the Illumina TruSeq TM and Nextera Mate Pair Library Preparation kits, respectively. All libraries were sequenced by LGC Genomics GmbH (Berlin, Germany) on an Illumina HiSeq 2000 platform using the paired-end protocol. Between 234 and 351 million DNA raw reads were generated per library (Table 1). Assembly and scaffolding of the cleaned reads were done with the software Allpaths-LG [14], followed by GapCloser for closing gaps in the assembled scaffolds. The resulting scaffolding was then further improved by integrating independent reconstructed transcript data after RNA sequencing (see Supplementary material for details). The OSCA v1.2 draft genome assembly consisted of 50,738 scaffolds, representing 419 Mb with a mean read coverage of 50 reads per base (Table 2). N50 and N90 were 29,308 and 3051 bp, respectively. Scaffold lengths ranged between 883 bp and 619.8 kb. The genome assembly had a GC content of 37.4% and a proportion of repeated elements of 16.6% (Table 3). A total of 8372 short duplicated regions (average length: 1813 bp, min.: 808 bp, max.: 11,745 bp) were identified on 7009 scaffolds. The quality of the OSCA v1.2 genome assembly was evaluated by the recovery rate of three sets of genes highly conserved in eukaryotes and arthropods [15], [16]. Among both eukaryotic gene sets, 61% of the CEGMA genes and 70% of the BUSCO genes were recovered in the OSCA v1.2 genome, while 51% of the BUSCO arthropod conserved genes could be identified. Using the MAKER pipeline [see Supplementary material, [17]] on the OSCA v1.2 nuclear genome 26,120 coding genes were predicted (Table 4). Of these coding genes, 80.3% could be functionally annotated. Furthermore, 19,023 OSCA genes were assigned to 9785 ortholog groups (Fig. 1) of which 93% were shared with at least one of the three Lepidoptera species *Bombyx mori*, *Danaus plexippus* or *Spodoptera frugiperda*.

**Table 1:** Statistic features of sequence reads issued from different genomic libraries and used for the OSCA v1.2 genome assembly.

**Library**	**PE300**	**Mate3kb**	**Mate8kb**
Raw read count	350,636,628	234,843,432	295,988,124
Read count after clipping^a^	349,359,410	233,657,586	287,067,720
Total Size [Gb]	34.5	11.9	23.9
Minimum Length [bp]	20	20	20
Maximum Length [bp]	100	51	100
Mean Length [bp]	99	51	83
Expected insert size [bp]^b^	300	2500	7500
Mapped on OSCA v1.2 genome	207,000,258	101,565,815	146,139,301
Mapped as paired-end on OSCA v1.2 genome	170,567,848	16,877,194	31,116,832

a Only paired-end reads taken into account

b Based on *in silico* measurements

**Table 2:** Genome features of the OSCA v1.2 assembly. The coverage is defined as the average read count per assembled bp.

	OSCA v1.2 assembly
Contig count	163,703
Scaffold (scf) count	50,738
N50 scf [bp]	29,308
N50 scf sequence count	3395
N90 scf [bp]	3051
N90 scf sequence count	21,872
Minimum scf length [bp]	883
Maximum scf length [kb]	619.8
Illumina PE300 coverage [reads/bp]	49.7
GC content [%]	37.4
N base content in assembly [%]	30.9
Total Length [Mb]	419.2
Total Length without Ns [Mb]	289.8
GenomeScope PE genome size estimation [Mb]	302.9
CEGMA identified [%] (count of 248)	152 (61.3)
CEGMA full-length [%] (count of 248)	83 (33.5)
BUSCO2 euk identified [%] (count of 303)	211 (69.6)
BUSCO2 euk full-length [%] (count of 303)	156 (51.5)
BUSCO2 arthropod identified [%] (count of 2675)	1363 (50.9)
BUSCO2 arthropod full-length [%] (count of 2675)	842 (31.5)

**Table 3:** Number of repeated elements found in the OSCA v1.2 draft genome assembly and corresponding genome ratio.

**Family**	**Fragments**	**Total length [Mb]**	**% of genome**
LTR	413,393	45.8	10.9
LINE	95,506	11.8	2.8
SINE	34,673	3.8	0.9
DNA	64,646	8	1.9
Total	608,218	69.4	16.6

**Table 4:** Characteristics of the different transcriptome assemblies and the genes predicted from the genome.

	HiSeq transcriptome	454 transcriptome	MAKER genes
Raw read count	325,008,948	322,504	*N/A*
Cleaned read count	267,359,188	287,429	*N/A*
Mapped Reads	198,962,467	145,588	*N/A*
Transcriptome size [Mb]	49.2	10.4	21.6
Coverage (mean read count per bp)	339.2	9.8	*N/A*
Transcript count	44,564	11,231	26,120
Unigene count	33,080	8892	26,120
Mean CDS/transcript length [bp]	1103	922	829
Median transcript length [bp]	591	693	498
N50 transcript length [bp]	2006	1036	1296
N50 sequence count	7061	2858	4475
N90 transcript length [bp]	429	485	353
N90 sequence count	26,722	8736	17,539
Minimum length	201	111	66
Maximum length	27,559	10,991	53,685
CEGMA identified [%] (count of 248)	209 (84.3)	56 (22.6)	*N/A*
CEGMA full-length [%] (count of 248)	189 (76.2)	22 (8.9)	*N/A*
BUSCO2 euk identified [%] (count of 303)	268 (88.4)	70 (23.1)	226 (74.6)
BUSCO2 euk full-length [%] (count of 303)	256 (84.5)	17 (5.6)	162 (53.5)
BUSCO2 arthropod identified [%] (count of 2675)	1892 (70.7)	265 (9.9)	1453 (54.3)
BUSCO2 arthropod full-length [%] (count of 2675)	2109 (78.8)	119 (4.4)	923 (34.5)
Transcripts with predicted CDS (%)	18,494 (41.5)	4016 (35.8)	26,120 (100)
Transcripts with full-length CDS (%)	12,515 (28.1)	1826 (16.3)	26,120 (100)
Located on OSCA v1.2 genome [count] (%)	11,010 (24.7)	3331 (29.7)	26,120 (100)
Split on two OSCA v1.2 scaffolds [count] (%)	11,721 (26.3)	2350 (20.9)	0 (0)

### 2.2 *De novo* transcriptome

In March 2011, diapausing larvae were collected in mugwort stems from Nadarzin (Poland) and then reared in the laboratory to obtain fresh tissues from the following developmental stages: eggs and larval whole body and hemolymph from the fifth instar. At the adult stage we sampled and separated heads/thorax from abdomens and males from females. In total, we prepared 7 RNA extracts corresponding to these various stages and tissues (Table 5). RNA quality and concentration were evaluated using the RNA 6000 Nano kit with an Agilent 2100 Bioanalyser (Agilent Technologies, Palo Alto, CA, USA). Indexed cDNA libraries with an insert size of 150–200 bp were constructed for each developmental stage and tissue extract using the Illumina TruSeq RNA sample preparation kit. Subsequently, the libraries were sequenced by an Illumina HiSeq 2000 System at GATC Biotech (Konstanz, Germany) using the paired-end protocol. Between 32 and 59 million 100 bp raw reads were generated per library (Table 5). After a series of read cleaning and normalization steps, transcripts were reconstructed with Trinity, CD-HIT-EST and CAP3 [see Supplementary material, [[18], [19], [20]]]. The *de novo* transcriptome assembly had an overall size of 49.2 Mb and resulted in 44,564 transcripts, grouped into 33,080 unigenes (Table 4). Transcript lengths ranged from 201 to 27,559 bp. The N50 and N90 lengths were 2006 and 429 bp, respectively. Regarding the conserved eukaryotic gene sets, 84% of the CEGMA and 88% of the BUSCO genes were identified. Furthermore, 71% of the conserved arthropod BUSCO genes were present within the reference transcriptome. Coding sequences (CDS) were predicted with FrameDP [21] for 18,494 transcripts of which 12,515 were complete. An additional analysis of the 26,070 transcripts without predicted CDS with FEELnc [22] identified 10,883 potential long non-coding RNAs and sequences for which the CDS was either too fragmented (*n* = 15,132) or not present at all (*n* = 55). Further comparative analysis of the reference transcripts with the Lepbase [23] reference protein set detected probable homologs for 20,835 transcripts. OHR analyzes [12] on the best matches indicated that 61% of the CDS were reconstructed at least at 60% of the corresponding reference lepidopteran protein homolog, whereas 43% of the transcript CDS were assembled at full length.

**Table 5:** Developmental stages and tissues of 7 RNA extracts issued from F1 individuals obtained in the laboratory after rearing diapausing larvae collected in the field.

**Extract/library ID**	**Developmental stage**	**Tissue**	***n***	**Raw read count**
Lib1	egg	whole egg	3 egg masses (*ca*. 60 eggs)	58,941,438
Lib2	L5	whole body	9	54,020,124
Lib3	L5	hemolymph	31	43,406,048
Lib4	Female adult	Head/thorax	4	43,465,884
Lib5	Female adult	Abdomen	4	54,635,026
Lib6	Male adult	Head/thorax	4	38,226,332
Lib7	Male adult	Abdomen	4	32,314,096
Total				325,008,948
