# Supplementary material for: De novo genome and transcriptome resources of the Adzuki bean borer Ostrinia scapulalis (Lepidoptera: Crambidae)
Source: Data Brief. 2018 Feb 1;17:781–7. doi: 10.1016/j.dib.2018.01.073 (PMC5958680; doi:10.1016/j.dib.2018.01.073)
Supplement: Supplementary file 2 — Supplementary material [file mmc2.pdf]

Supplementary Material to the Data article entitled:

De novo genome and transcriptome resources of the Adzuki bean borer *Ostrinia scapulalis* (Lepidoptera: Crambidae)

Authors: B. Gschloessl, F. Dorkeld, P. Audiot, A. Bretaudeau, C. Kerdelhué and R. Streiff

De novo O. scapulalis reference transcriptome

1.1. *De novo* assembly of HiSeq transcriptome

The FASTQ reads of 7 Hiseq RNAseq paired-end libraries were combined to generate a *de novo O. scapulalis* reference transcriptome. Quality control of the raw Illumina reads was achieved with the FastQC program [v0.11.2, 1]. Trimomatic [v0.25, 2] was run with the following parameters: 2:40:15 HEADCROP:12 SLIDINGWINDOW:4:15 MINLEN:30, to eliminate low quality reads and bases, and to trim the adapter sequences. Poly A and poly T tails longer than 5 bp were removed by prinseq [v0.20.2, 3] (parameters : -trim_tail_left 5 -trim_tail_right 5 -min_len 30 -out_format 3). A total of 133,679,594 read pairs were retained after this filtering process and were used for transcriptome reconstruction with Trinity [vr2013_08_14, 4]. The normalization option (--normalize_reads) of Trinity was activated to optimize the computation time. Transcripts longer than 200 bp were retained. This procedure yielded 108,445 transcripts representing 68,902 unigenes. Additional steps were conducted to finalize the reference transcriptome: first, transcripts were clustered by CD-HIT-EST [CD-HIT package, v4.5.4, 5] with an identity threshold of 98% (parameters: -c 0.98 -l 20 -M 0 -B 1), and the longest sequence was retained for each cluster. Second, overlapping

transcripts were assembled with CAP3 [v2015/10/02, 6] (parameters: -o 80 -p 90) to obtain longer sequences. The resulting transcripts were filtered to remove poorly covered sequences using scripts of the Trinity package: cleaned RNAseq reads were aligned against the CAP3 transcripts; then, transcripts were filtered on their abundance with the RSEM program [RNA-Seq by Expectation Maximization, v1.2.8, default parameters, 7] which was run by the tool *align_and_estimate_abundance.pl* (--est_method RSEM) embedded in the Trinity package. Each read library was realigned on the reconstructed transcripts with Bowtie [v2.2.4, default parameters and and --reorder, 8]. Guided by these alignments, RSEM estimated the abundance of each transcript. For this purpose, the metric, `fragments per kilobase transcript length per million fragments mapped` (FPKM) was chosen to further exclude transcripts with low coverage (*i.e*. FPKM ≤ 2). The final set of 44,564 transcripts constituted the *O. scapulalis* reference transcriptome.

1.2. Quality assessment of the *de novo* reference transcriptome

The completeness of the *de novo* reference transcriptome was assessed with the CEGMA program [v2.5, default parameters, 9] which searched 248 orthologous groups of eukaryotic proteins and the BUSCO software [v2.0, 10] which was separately run (common parameters -m genome --long -f) to search 303 conserved eukaryotic genes (parameters: --lineage eukaryote -m trans) and 2,675 conserved arthropod genes (parameters: --lineage arthropoda -m trans). Coding sequences (CDS) within the reference transcripts were predicted with FrameDP [v1.2.2, default parameters, 11]. Furthermore, the FEELnc tool *FEELnc_codplot.pl* [vApril 2015, 12] was run to identify potential long non-coding RNAs and mRNAs with highly fragmented and short CDS. In detail, the transcript sequences without any FrameDP CDS were given as candidate file (-i), those sequences with predicted CDS were applied as annotated mRNAs (-a) and the `shuffle` mode was run as described in the FEELnc program documentation.

The ortholog hit ratio [OHR, 13] was calculated as an additional quality assessment of the reference transcripts. The OHR estimates the completeness of an assembled transcript based on the ratio of the number of bases in the *blastx* alignment of a *de novo* transcript compared to the length of the best match within a protein reference set. The *de novo* transcripts were aligned with *blastx* [v2.2.28, parameters: outfmt 5 -evalue 1e-5 -gapopen 11 -gapextend 1 -word_size 3 -matrix BLOSUM62; 14] against a reference set corresponding to 471,938 protein sequences extracted from the lepidopteran genome database Lepbase [v4, 15]. The OHR values were then calculated on the best hit sequence as described in Gschloessl, et al. [16].

The paired-end RNAseq shotgun reads were aligned with Bowtie (v2.2.4, default parameters and --reorder) on the *de novo* transcripts to estimate the transcript coverage. Only read pairs with mapping distances consistent with the specific library insert size were retained by applying the options --no-discordant and --maxins 500. The percentage of aligned reads and the read per bp coverage were calculated for each transcript using SAMtools [v1.2, 17], BAMtools [v2.3.0, 18] and BEDtools [v2.2.23, 19].

2. *De novo O. scapulalis* genome

2.1. Bioinformatics analysis and LGC assembly protocol

Sequencing and read quality cleaning

The genomic libraries were sequenced (30 May 2013) with Illumina HiSeq2000 technology. The shotgun paired-end library (1 Illumina lane) was sequenced as 2x100 bp. The reads were post-processed with the CASAVA program (v1.8.2, https://support.illumina.com/content/dam/illumina-support/documents/myillumina/33d66b02-53b5-4f4d-9d8b-f94237c7e44d/casava_qrg_15011197b.pdf) to generate demultiplexed FASTQ files. Sequencing adapters (Illumina TruSeq and Cre-LoxP mate pair linkers) were clipped and quality trimming was

applied on all reads. More precisely, reads containing more than one N were removed. Base pairs or complete reads with sequencing errors, which occur on Illumina reads mainly at the 3'-end, were also filtered. Thus, further quality trimming of the 3' read end was done to obtain a minimum average Phred quality score of 10 over a window of ten bases. A minimum read length of 20 bp was requested. Error correction of the quality-trimmed reads was achieved using Musket [v1.0.6, 20] with a k-mer size of 21. Then, digital normalization of error-corrected reads (folder 'DigitallyNormalized') was conducted with the program *normalize_by_median.py* from the 'khmer' package [v0.3, 21] applying a k-mer size of 20 and a coverage cutoff of 80. Reads with final length shorter than 21 bases were discarded. Only properly paired reads were retained for the genome assembly.

Draft genome assembly OSCA *v1.1*

Two genome assemblies and scaffoldings were conducted by LGC using Allpaths-LG [release 47547, 22]. The first allowed identifying and filtering mate pair reads with unexpected insert sizes: all mate pair reads were aligned with Bowtie (v2.1.0, default parameters) on this first assembly and only mate pair reads properly paired with a consistent insert size [4,500-7,500 bp], dangling mate pairs (only one mate mapped) and non-mapping mate pairs were retained. Then, a second assembly and scaffolding were run including these filtered mate pair reads and properly-paired shotgun reads. Gaps were closed in the second assembly - named OSCA v1.1 - using SOAP GapCloser (v1.12, http://soap.genomics.org.cn/soapdenovo.html) with a k-mer size of 31 and all digitally normalized, error-corrected reads as input. Finally, putative mitochondrial scaffolds were filtered from the nuclear genome assembly using *blastn* on the OSCA v1.1 scaffolds against the *Ostrinia furnacalis* mitochondrion sequence (NCBI accession number NC_003368.1).

OSCA v1.2: Integrating transcriptomic data to improve the scaffolding of the draft genome assembly

The *de novo* reference transcriptome, two other independent *O. scapulalis* transcript sets (NCBI BioProjects PRJNA392376 and PRJNA396660; Orsucci et al. submitted), and a previously published *O. scapulalis* 454 transcript set [16] were integrated into the scaffolding process to further improve the OSCA v1.1 scaffolding. To do so, sub-sequences of 500 bp length (parameter –size 500) and 300 bp overlap (parameter –overlap 300) were generated from all available 63,471 transcript sequences by applying the *splitter* program of the EMBOSS bioinformatics tools package [v6.4.0.0, 23]. In total 493,704 pseudo long reads were obtained and were aligned with BLAT [v35, parameters: -t=dna -q=dna -noHead; 24] on the OSCA v1.1 genome scaffolds. Then, the program L_RNA_scaffolder [downloaded 2016/06/17, default parameters, 25] used the information from the transcript alignments (in PSL format) to identify overlaps between genome scaffolds. The resulting genome assembly was called OSCA v1.2.

2.2. Characteristics and quality assessment of the OSCA v1.2 genome assembly

The GC content and statistical features (N50, N90, mean, *etc.*) of the OSCA v1.2 genome assembly were calculated with in-house Perl scripts. Repeated elements in the draft genome assembly were identified with RepeatModeler [v1.0.8, 26] (parameter: -engine ncbi) and RepeatMasker [v4.0.6, 27] (parameters: -s xsmall -gff –norna –engine ncbi –poly –gff). The results of RepeatMasker were summarized by the tool "One code to find them all" [v03/2016, 28] applying default parameters. The OSCA v1.2 read per base coverage and the remapping percentage were calculated for each genome scaffold as described above (see reference transcriptome section). In addition, the *in silico* expected full genome size of the OSCA v1.2 genome was calculated by counting the frequencies of k-mers of 21 bp length within all Illumina paired-end raw reads with the software Jellyfish [v1.1.11, 29] and the web-software GenomeScope [parameters: kmer=21, max cov=150, read length=100bp; 30]. The

expected full genome size for *O. scapulalis* was estimated to be ca. 303 Mb, which is shorter than the OSCA v1.2 assembly size.

In addition, the quality of the OSCA v1.2 genome assembly was assessed by screening duplicated regions within each scaffold. Each scaffold was aligned against itself by global alignment with LASTZ [v1.03.02, 31] applying the following parameters: --notransition --step=20 --gfextend --gapped --chain --matchcount=800 --identity=92 --format=general:score,name1,zstart1,end1,strand1,size1,name2,zstart2+, end2+,strand2,size2.

Next, well-conserved core genes were searched for in the OSCA v1.2 draft genome with CEGMA (default parameters) and BUSCO (common parameters: -m genome --long -f; eukaryotic set: --lineage eukaryote; arthropoda set: --lineage arthropoda) in order to obtain an estimate of the assembly completeness. Last, the CEGMA and BUSCO protein sequences that had not been identified in the OSCA v1.2 assembly were aligned with *tblastn* (NCBI-BLAST+ v2.2.29; parameters: -evalue 1e-5 –outfmt 7 –num_alignments 20) to the OSCA v1.2 assembly in order to retrieve the sequences that were split on two *O. scapulalis* genome scaffolds. The corresponding core genes were declared as being present in the assembly if the alignment covered at least 40% of the KOG.

2.3. MAKER structural annotation

The structural annotation of OSCA v1.2 was obtained by running the gene prediction program MAKER [v2.31.9, 32] in an iterative manner as suggested by Campbell, et al. [33]. Precisely, the MAKER output (Maker1.1) was given in GFF3 format as MAKER annotation to a second MAKER gene prediction (Maker1.2) in order to refine the structural annotation. In the following the procedure is listed in more detail. Several parameters and input files (*maker_opts.ctl* configuration file) were common to both MAKER runs (Maker1.1 and 1.2): (i) the OSCA v1.2 genome assembly file was given

as ‘genome’. (ii) As ‘est’ evidence, four *O. scapulalis* transcript sets were merged into one file totalizing 99,393 transcript sequences, namely the *de novo* transcripts described in the present manuscript, the 454 transcripts described in Gschloessl, et al. [16] and two additional *O. scapulalis* transcript sets (Orsucci et al., submitted). (iii) 471,938 protein sequences obtained from Lepbase (v4) were given as ‘protein’ homology evidence. (iv) The gene prediction model *tribolium2012* was chosen as ‘augustus_species’.

Particular features of the Maker1.1 run were the ‘snaphmm’ gene model for which the *cegma.hmm outputfile of the CEGMA analysis run on the OSCA v1.2 genome assembly was applied and the use of EST (est2genome=1) and protein evidences (protein2genome=1) to infer gene predictions directly. For the Maker1.2 run, the Maker1.1 predictions were extracted as GFF3 file (*gff3_merge*) and provided as input (parameter maker_gff). Additionally, the same GFF3 file was used to generate a new SNAP model (MAKER tool *maker2zff*) which was given as MAKER parameter ‘*snaphmm*’. The direct inference of gene predictions from EST (est2genome=0) and protein evidences (protein2genome=0) was deactivated. The Maker1.2 predictions were post-processed to generate a GFF3 file (*fasta_merge* and awk to retain ‘maker’-specific output) and to add species-specific IDs to the predicted gene structures (tools *maker_map_ids* and *map_gff_ids*). Then, the cufflinks [v2.2.1, 34] *gffread* tool was run on the created GFF3 file to generate FASTA files for mRNAs (-w), CDS (-x) and proteins (-y). Finally, a BUSCO search for conserved eukaryotic and arthropod genes was conducted on the encoded transcripts of the predicted gene set, applying the same parameters as for reference transcriptome analysis.

3. Reference transcripts in the OSCA v1.2 genome and functional annotation

Mapping the reference transcripts onto the OSCA v1.2 *assembly*

The *de novo* transcripts and the published 454 resource [16] obtained from the same population were aligned to the *de novo* genome using BLAT [v35, default parameters, 24]. An in-house Perl script was used to select only well-aligned transcripts (at least 80% of the transcript aligned with 92% identity, as proposed by Stanke [35]). The same procedure as previously used to identify KOG genes split over two scaffold ends was applied to reference and 454 transcripts, except that the *blastn* parameters -soft_masking true –evalue 1e-5 –outfmt 7 –show_gis –perc_identity 70 were used to align the nucleotide transcript sequences against the OSCA v1.2 assembly.

3.1. Functional annotation of MAKER genes and reference transcriptome

The MAKER genes as well as the *de novo* assembled reference transcripts with predicted CDS were functionally annotated via an automated procedure. To do so, for each set the longest encoded proteins to each gene/transcript were aligned against the NCBI NR database (v06/05/2017) with *blastp* (v2.5.0+ minimum e-value=1e-8, maximum target sequences=20). An InterProScan [v5.13-52.0, 36] analysis was conducted on the same dataset. Then, Blast2GO [v2.5, database vOct2016, 37] assigned Gene Ontology (GO) terms to proteins, taking into account the *blastp* and InterProScan results.

3.2. Identification of orthologs with other insect species

The OrthoMCL package [blastp v2.5.0+, parameters: -evalue 1e-5; orthomcl v2.0.9 parameters: percentMatchCutoff=50 evalueExponentCutoff=-5, mcl v14-137 with parameters: --abc -I 1.5; 38] was run to identify potential orthologs between the FrameDP-predicted proteins corresponding to

the *de novo* transcripts and the protein sets of *Drosophila melanogaster* (version dmel-all-translation-r6.03), *Danaus plexippus* (version Danaus_plexippus.DanPle_1.0.25), *Bombyx mori* (version http://sgp.dna.affrc.go.jp/ComprehensiveGeneSet) and the noctuoid *Spodoptera frugiperda*. For this latter species, proteins-coding sequences (CDS) were identified by FrameDP (default parameters) on the transcript set [39] obtained from LepidoDB (http://bipaa.genouest.org/data/public/lepidodb/TR2012b.fa). For each of the 22,253 *S. frugiperda* transcripts with predicted CDS, the longest encoded protein was included into the OrthoMCL orthology analysis. Results were visualized as a Venn diagram using the R package VennDiagramm [v1.6.17 , 40].

4. LepidoDB

The genomic and transcriptomic resources developed in the present study were integrated into LepidoDB (http://bipaa.genouest.org/sp/ostrinia_scapulalis/). All predicted MAKER genes and the reference transcripts along with their corresponding functional annotations were loaded into a Chado database [v1.31, 41]. All data available for each transcript were gathered in specific web pages using Tripal [v2.1, 42]. For visualization purposes, a JBrowse [v1.12.3, 43] genome browser was set up. To facilitate analyses of the *O. scapulalis* resource data, a search engine, a BLAST and a Galaxy server [44] can be accessed from the web page.

REFERENCES

[1] S. Andrews, FastQC: A Quality Control tool for High Throughput Sequence Data, in: Software, http://www.bioinformatics.babraham.ac.uk/projects/fastqc/.
[2] A.M. Bolger, et al., Trimmomatic: A flexible trimmer for Illumina sequence data, Bioinformatics, 30 (2014) 2114-2120.
[3] R. Schmieder, R. Edwards, Quality control and preprocessing of metagenomic datasets, Bioinformatics, 27 (2011) 863-864.
[4] M.G. Grabherr, et al., Full-length transcriptome assembly from RNA-Seq data without a reference genome, Nature Biotechnology, 29 (2011) 644-652.
[5] L. Fu, et al., CD-HIT: Accelerated for clustering the next-generation sequencing data, Bioinformatics, 28 (2012) 3150-3152.
[6] X. Huang, A. Madan, CAP3: A DNA sequence assembly program, Genome Res., 9 (1999) 868-877.
[7] B. Li, C.N. Dewey, RSEM: Accurate transcript quantification from RNA-Seq data with or without a reference genome, BMC Bioinformatics, 12 (2011).
[8] B. Langmead, S.L. Salzberg, Fast gapped-read alignment with Bowtie 2, Nat. Methods, 9 (2012) 357-359.
[9] G. Parra, et al., CEGMA: A pipeline to accurately annotate core genes in eukaryotic genomes, Bioinformatics, 23 (2007) 1061-1067.
[10] F.A. Simao, et al., BUSCO: Assessing genome assembly and annotation completeness with single-copy orthologs, Bioinformatics, 31 (2015) 3210-3212.
[11] J. Gouzy, et al., FrameDP: Sensitive peptide detection on noisy matured sequences, Bioinformatics, 25 (2009) 670-671.
[12] V. Wucher, et al., FEELnc: a tool for long non-coding RNA annotation and its application to the dog transcriptome, Nucleic Acids Res., 45 (2017) e57.
[13] S.T. O'Neil, et al., Population-level transcriptome sequencing of nonmodel organisms *Erynnis propertius* and *Papilio zelicaon*, BMC Genomics, 11 (2010).
[14] S.F. Altschul, et al., Basic local alignment search tool, J Mol Biol, 215 (1990) 403-410.
[15] R.J. Challis, et al., Lepbase: The Lepidopteran genome database, bioRxiv, (2016).
[16] B. Gschloessl, et al., *De novo* transcriptomic resources for two sibling species of moths: *Ostrinia nubilalis* and *O. scapulalis*, BMC Res Notes, 6 (2013).
[17] H. Li, et al., The Sequence Alignment/Map format and SAMtools, Bioinformatics, 25 (2009) 2078-2079.
[18] D.W. Barnett, et al., BamTools: A C++ API and toolkit for analyzing and managing BAM files, Bioinformatics, 27 (2011) 1691-1692.
[19] A.R. Quinlan, BEDTools: The Swiss-Army Tool for Genome Feature Analysis, Current Protocols in Bioinformatics, 47 (2014) 1-34.
[20] Y. Liu, et al., Musket: a multistage k-mer spectrum-based error corrector for Illumina sequence data, Bioinformatics, 29 (2013) 308-315.
[21] M.R. Crusoe, et al., The khmer software package: enabling efficient nucleotide sequence analysis, F1000Research, 4 (2015) 900.
[22] S. Gnerre, et al., High-quality draft assemblies of mammalian genomes from massively parallel sequence data, Proc. Natl. Acad. Sci. U.S.A, 108 (2011) 1513-1518.
[23] P. Rice, et al., EMBOSS: the European Molecular Biology Open Software Suite, Trends Genet, 16 (2000) 276-277.
[24] W.J. Kent, BLAT--the BLAST-like alignment tool, Genome Research, 12 (2002) 656-664.
[25] W. Xue, et al., L_RNA_scaffolder: Scaffolding genomes with transcripts, BMC Genomics, 14 (2013).
[26] A.F.A. Smit, R. Hubley, RepeatModeler in: Software, http://www.repeatmasker.org, 2008-2015.
[27] A.F.A. Smit, et al., RepeatMasker in: Software, http://www.repeatmasker.org, 2013-2015.

[28] M. Bailly-Bechet, et al., “One code to find them all”: A perl tool to conveniently parse RepeatMasker output files, Mobile DNA, 5 (2014).

[29] G. Marcais, C. Kingsford, A fast, lock-free approach for efficient parallel counting of occurrences of k-mers, Bioinformatics, 27 (2011) 764-770.

[30] G.W. Vurture, et al., GenomeScope: Fast reference-free genome profiling from short reads, Bioinformatics, (2017).

[31] R.S. Harris, Improved pairwise alignment of genomic DNA., in: Computer Science and Engineering, The Pennsylvania State University, USA, 2007, pp. http://www.bx.psu.edu/~rsharris/rsharris_phd_thesis_2007.pdf.

[32] B.L. Cantarel, et al., MAKER: An easy-to-use annotation pipeline designed for emerging model organism genomes, Genome Res., 18 (2008) 188-196.

[33] M.S. Campbell, et al., Genome Annotation and Curation Using MAKER and MAKER-P, Curr Protoc Bioinformatics, 48 (2014) 4.11.11-39.

[34] C. Trapnell, et al., Transcript assembly and quantification by RNA-Seq reveals unannotated transcripts and isoform switching during cell differentiation, Nat. Biotechnol., 28 (2010) 511-515.

[35] M. Stanke, Incorporating RNA-Seq into AUGUSTUS, in, 2009.

[36] E.M. Zdobnov, R. Apweiler, InterProScan--an integration platform for the signature-recognition methods in InterPro, Bioinformatics, 17 (2001) 847-848.

[37] A. Conesa, et al., Blast2GO: A universal tool for annotation, visualization and analysis in functional genomics research, Bioinformatics, 21 (2005) 3674-3676.

[38] L. Li, et al., OrthoMCL: Identification of ortholog groups for eukaryotic genomes, Genome Res., 13 (2003) 2178-2189.

[39] F. Legeai, et al., Establishment and analysis of a reference transcriptome for *Spodoptera frugiperda*, BMC Genomics, 15 (2014).

[40] H. Chen, P.C. Boutros, VennDiagram: A package for the generation of highly-customizable Venn and Euler diagrams in R, BMC Bioinformatics, 12 (2011).

[41] C.J. Mungall, D.B. Emmert, A Chado case study: An ontology-based modular schema for representing genome-associated biological information, Bioinformatics, 23 (2007) i337-i346.

[42] L.A. Sanderson, et al., Tripal v1.1: a standards-based toolkit for construction of online genetic and genomic databases, Database (Oxford), 2013 (2013) bat075.

[43] M.E. Skinner, et al., JBrowse: A next-generation genome browser, Genome Research, 19 (2009) 1630-1638.

[44] D. Blankenberg, et al., Galaxy: A web-based genome analysis tool for experimentalists, Curr Protoc Mol Biol, 19 (2010).
